# Longitudinal study of epigenetic aging and its relationship with brain aging and cognitive skills in young adulthood

**DOI:** 10.3389/fnagi.2023.1215957

**Published:** 2023-08-01

**Authors:** Klara Mareckova, Anna Pacinkova, Radek Marecek, Ludmila Sebejova, Lydie Izakovicova Holla, Jana Klanova, Milan Brazdil, Yuliya S. Nikolova

**Affiliations:** ^1^Brain and Mind Research, Central European Institute of Technology (CEITEC), Masaryk University (MU), Brno, Czechia; ^2^1^St^ Department of Neurology, St. Anne’s University Hospital and Faculty of Medicine, Masaryk University, Brno, Czechia; ^3^Faculty of Informatics, Masaryk University, Brno, Czechia; ^4^RECETOX, Faculty of Science, Masaryk University, Brno, Czechia; ^5^Department of Stomatology, St. Anne’s Univ. Hospital and Faculty of Medicine, Masaryk University, Brno, Czechia; ^6^Campbell Family Mental Health Research Institute, Centre for Addiction and Mental Health (CAMH), Toronto, ON, Canada; ^7^Department of Psychiatry, University of Toronto, Toronto, ON, Canada

**Keywords:** epigenetic age, brain age, IQ, longitudinal, magnetic resonance imaging (MRI)

## Abstract

**Introduction:**

The proportion of older adults within society is sharply increasing and a better understanding of how we age starts to be critical. However, given the paucity of longitudinal studies with both neuroimaging and epigenetic data, it remains largely unknown whether the speed of the epigenetic clock changes over the life course and whether any such changes are proportional to changes in brain aging and cognitive skills. To fill these knowledge gaps, we conducted a longitudinal study of a prenatal birth cohort, studied epigenetic aging across adolescence and young adulthood, and evaluated its relationship with brain aging and cognitive outcomes.

**Methods:**

DNA methylation was assessed using the Illumina EPIC Platform in adolescence, early and late 20 s, DNA methylation age was estimated using Horvath’s epigenetic clock, and epigenetic age gap (EpiAGE) was calculated as DNA methylation age residualized for batch, chronological age and the proportion of epithelial cells. Structural magnetic resonance imaging (MRI) was acquired in both the early 20 s and late 20 s using the same 3T Prisma MRI scanner and brain age was calculated using the Neuroanatomical Age Prediction using R (NAPR) platform. Cognitive skills were assessed using the Wechsler Adult Intelligence Scale (WAIS) in the late 20 s.

**Results:**

The EpiAGE in adolescence, the early 20 s, and the late 20 s were positively correlated (*r* = 0.34–0.47), suggesting that EpiAGE is a relatively stable characteristic of an individual. Further, a faster pace of aging between the measurements was positively correlated with EpiAGE at the end of the period (*r* = 0.48–0.77) but negatively correlated with EpiAGE at the earlier time point (*r* = −0.42 to −0.55), suggesting a compensatory mechanism where late matures might be catching up with the early matures. Finally, higher positive EpiAGE showed small (Adj *R*^2^ = 0.03) but significant relationships with a higher positive brain age gap in all participants and lower full-scale IQ in young adult women in the late 20 s.

**Discussion:**

We conclude that the EpiAGE is a relatively stable characteristic of an individual across adolescence and early adulthood, but that it shows only a small relationship with accelerated brain aging and a women-specific relationship with worse performance IQ.

## 1. Introduction

While there were only 5% of the world population over 65 years old in 1950, it is approximately 9% today and this proportion is predicted to increase to 17% by 2050 (He et al., 2016). Given this sharp increase in the proportion of older adults within society, a better understanding of how we age starts to be critical. It has been demonstrated that the pace of biological aging varies between people, independently of chronological age (Belsky et al., 2015; Beltrán-Sánchez et al., 2022; Jáni et al., 2022; Mareckova et al., 2020a; Marečková et al., 2020b). However, longitudinal studies are needed to elucidate why some people experience accelerated and others decelerated biological aging and how the speed of biological aging relates to brain health and cognitive skills.

Since DNA methylation patterns change predictably over time and are highly correlated with age, DNA methylation patterns can be used to predict one’s chronological age. The two most commonly used DNA methylation-based predictors of age are the multi-tissue Horvath’s epigenetic clock (Horvath, 2013) and the blood-based Hannum’s epigenetic clock (Hannum et al., 2013). Further research demonstrated that accelerated epigenetic aging, defined as the residual variation in epigenetic age independent of chronological age, is linked with decreased physical capability and cognitive functioning (Jain et al., 2022) as well as male sex and clinical traits such as greater risk for cardiovascular disease or diabetes (Oblak et al., 2021). In contrast, decelerated epigenetic aging is characteristic of long-lived individuals.

According to twin studies, the heritability of epigenetic age acceleration is relatively high (*h*^2^ ∼ 40%; Horvath and Raj, 2018). Further research (Horvath, 2013) suggested that the speed of the epigenetic clock might be more heritable at a younger age but less heritable later in life when the environmental contribution to epigenetic aging increases. However, it is not clear how epigenetic aging relates to brain aging and cognition. While Sanders et al. (2022) did not find any associations between methylation age, brain age, and cognitive abilities in late adolescence, Zhou et al. (2022) did find relationships between epigenetic and brain aging in midlife and pointed out that both faster epigenetic and brain aging were associated with worse cognition. These studies suggest that the relationships among epigenetic age assessments as well as the relationships among epigenetic age, brain age, and cognition may vary across the lifespan.

Cross-sectional studies cannot directly assess the dynamics of aging and longitudinal studies following an individual over decades are needed to provide actionable insight into the aging process. Moreover, in order to identify early markers of accelerated aging and cognitive decline, and direct early interventions accordingly, these longitudinal studies should start following its participants before the emergence of overt symptoms of cognitive deterioration. However, given the paucity of longitudinal studies with both neuroimaging and epigenetic data, it remains largely unknown whether the speed of the epigenetic clock changes over the life course and whether any such changes are proportional to, or independent of, changes in brain aging and cognitive skills.

To address these knowledge gaps, the current study used longitudinal data from a prenatal birth cohort to assess epigenetic aging and its relationship with brain aging and cognitive outcomes. We aimed to answer three main research questions: (1) Does the epigenetic age gap change with time or is it a stable characteristic?, (2) Does accelerated epigenetic aging predict accelerated brain aging or are these independent?, (3) Does accelerated epigenetic aging predict deficits in cognition as early as young adulthood? Based on the literature reviewed above, we hypothesized that the epigenetic age gaps will be more strongly correlated at the younger age than at the older age, and that a greater positive epigenetic age gap will predict a greater positive brain age gap and worse cognitive skills.

## 2. Materials and methods

### 2.1. Participants

Participants were members of the European Longitudinal Study of Pregnancy and Childhood (ELSPAC; Golding, 1989; Piler et al., 2017), a prenatal birth cohort born between 1991 and 1992 in South Moravia, Czechia, who also participated in its two neuroimaging and epigenetics follow-ups: (1) Biomarkers and Underlying Mechanisms of Vulnerability to Depression (VULDE) and (2) Health Brain Age (HBA) at the Central European Institute of Technology, Masaryk University. All participants provided written informed consents to participate in the HBA and VULDE studies, including the agreement to merge data from HBA, VULDE, and their historical data from ELSPAC. Ethical approval for both the HBA and VULDE studies was obtained from the ELSPAC ethics committee.

### 2.2. DNA methylation and calculation of epigenetic age gap

Buccal swabs were collected from 261 participants in their late 20 s (*M* = 29.49 years, SD = 0.64). A subset of these participants had also buccal swabs from their early 20 s (*n* = 76; *M* = 23.85 years, SD = 0.39) and saliva samples from adolescence (*n* = 39; *M* = 14.65 years, SD = 0.84). DNA methylation from all these participants and time points was assessed using the Illumina EPIC Platform and “Methylation age” was estimated using the Horvath’s epigenetic clock (Horvath, 2013) as described in Marečková et al. (2020b). Briefly, R package ChAMP (Tian et al., 2017) was used to process the raw Illumina microarray data. Raw data were trimmed of (1) probes with <3 beads in at least 5% of samples per probe, (2) SNP-related probes, (3) multi-hit probes, (4) probes located in chromosomes X and Y. Beta mixture quantile normalization (BMIQ; Teschendorff et al., 2013) method was used to adjust the beta-values of type II design probes into a statistical distribution characteristic of type I probes. Next, DNA methylation age was calculated using an epigenetic clock developed by Horvath (2013), which uses 353 CpG sites to estimate DNA methylation age. Next, we residualized the DNA methylation age estimates at each timepoint for batch, chronological age, and the proportion of epithelial cells (the average proportion was 78% of epithelial and 22% of immune cells; SD = 22% in each group) in each participant and saved the residuals from the analysis as the epigenetic age gap (EpiAGE). Thus, positive values of EpiAGE reflect accelerated aging/faster maturation and negative values reflect decelerated aging/slower maturation. Pace of aging between the different time points was calculated as the difference between the respective EpiAGE variables. Sample size at the different time points is illustrated in Supplementary Figure 1 and the respective demographic information is provided in Supplementary Table 1.

### 2.3. Magnetic resonance imaging and calculation of brain age

Structural magnetic resonance imaging (MRI) was acquired in both the early 20 s and late 20 s using the same 3T Prisma MRI scanner. There were 261 participants at the second neuroimaging follow-up in the late 20 s (HBA study). A subset of these (*n* = 110) also participated in the first neuroimaging follow-up in the early 20 s (VULDE study). The brain age at both time points was calculated as described in Mareckova et al. (2023). Briefly, T1-weighted data were processed using FreeSurfer version 7.1.1 and the outputs were visually inspected for common artifacts (e.g., skull strip failure, spikes, parcellation issues, faulty gray and white matter boundaries). All participants passed these quality control procedures. Next, the Neuroanatomical Age Prediction using R (NAPR; Pardoe and Kuzniecky, 2018) platform was used to calculate participants’ brain age. The NAPR platform is a cloud-based tool (Amazon Web Services) that estimates the age of an individual using cortical thickness maps derived from their own locally processed T1-weighted whole-brain MRI scans (Pardoe and Kuzniecky, 2018). This age estimation model was trained on data from 2,367 healthy control participants from ages 6 to 89 years using relevance vector machine regression (Tipping, 2001) and Gaussian processes machine learning methods (Rasmussen and Williams, 2005) applied to cortical thickness surfaces obtained using FreeSurfer. Finally, BrainAGE was calculated as the difference between this cortical thickness-based estimate of brain age and each participant’s chronological age.

### 2.4. Assessment of cognition (IQ)

Cognitive ability was assessed using the seven-subtest short form of the Wechsler Adult Intelligence Scale (WAIS), fourth edition (Tam, 2004) in the late 20 s. This measure allowed a generation of performance IQ (subtests picture completion, digit-symbol coding, and matrix reasoning), verbal IQ (subtests information, arithmetic, similarity and digit span) and full-scale IQ.

### 2.5. Statistical analyses

All statistical analyses were performed in JMP version 10.0.0 (SAS Institute Inc., Cary, NC). First, we used Levene’s test to evaluate the equality of variance of EpiAGE at the three different time points (adolescence, early 20 s, late 20 s). Second, we assessed the Pearson correlations between EpiAGE in adolescence, early 20 s and late 20 s. Third, we assessed the Pearson correlations between the pace of epigenetic aging between (1) adolescence and early 20 s, (2) early 20 s and late 20 s, and (3) adolescence and late 20 s. And fourth, we assessed the Pearson correlations between the EpiAGE and pace of epigenetic aging measures. The possible effect of sex on EpiAGE and pace of aging at the different timepoints was evaluated using a t-test. Subsequently, we used a full-factorial general linear model (GLM) to evaluate the relationship between (1) EpiAGE, BrainAGE, and sex; and (2) EpiAGE, sex, and full-scale IQ in the late 20 s. Follow-up analyses then determined the role of verbal and performance IQ in these relationships.

## 3. Results

### 3.1. Do the epigenetic age gap and the pace of epigenetic aging change with time or are they a stable characteristic?

**FIGURE 1:**
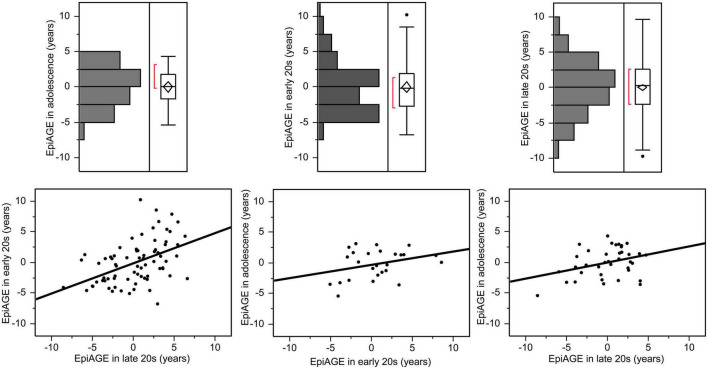
Epigenetic age gap estimates (EpiAGE) in adolescence, the early 20 s and the late 20 s and their correlations. The correlation between EpiAGE in the late 20 s and EpiAGE in the early 20 s was large (*r* = 0.47, *p* < 0.0001), the correlation between EpiAGE in the late 20 s and EpiAGE in adolescence was medium (*r* = 0.34, *p* = 0.038), and the correlation between the EpiAGE in the early 20 s and EpiAGE in adolescence did not reach significance (*r* = 0.32, *p* = 0.115).

The variance of EpiAGE at the three timepoints was not equal [*F*_(2,372)_ = 3.94, *p* = 0.02]. It increased with age and ranged from −5.40 to +4.34 years in adolescence, from −6.75 to +10.25 years in the early 20 s, and from −9.75 to +9.73 years in the late 20 s. The correlation between the closest measurements, namely between EpiAGE in the late 20 s and EpiAGE in the early 20 s, was large (*r* = 0.47, *p* < 0.0001). The correlation between the more distant measurements were medium (EpiAGE in the late 20 s and EpiAGE in adolescence: *r* = 0.34, *p* = 0.038). The correlation between the EpiAGE in the early 20 s and EpiAGE in adolescence did not reach significance (*r* = 0.32, *p* = 0.115), most likely due to the smaller sample size of these measurements. The distributions of EpiAGE at the three time points and the correlations between the measurements are illustrated in Figure 1.

**FIGURE 2:**
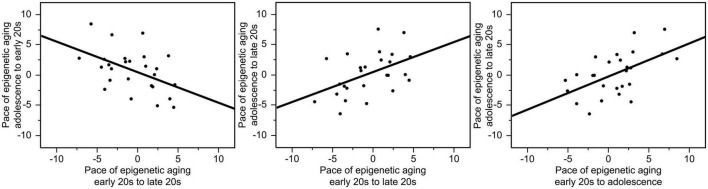
Correlations between the pace of epigenetic aging during these three different periods. Faster pace of epigenetic aging between the early and the late 20 s showed a negative relationship with the pace of epigenetic aging between adolescence and early 20 s (*r* = –0.48, *p* = 0.014). In contrast, faster epigenetic aging between adolescence and late 20 s predicted faster epigenetic aging between adolescence and early 20 s (*r* = 0.55, *p* = 0.004) as well as between the early and late 20 s (*r* = 0.47, *p* = 0.015).

The pace of epigenetic aging between the early 20 s and late 20 s varied from −9.37 to +9.73 years, the pace of epigenetic aging between adolescence and the early 20 s varied from −5.32 to +8.46 years, and the pace of epigenetic aging between adolescence and the late 20 s varied from −6.41 to +7.59 years. The correlations between the pace of epigenetic aging during these three different periods were high. Interestingly, faster pace of epigenetic aging between early and late 20 s showed a negative relationship with the pace of epigenetic aging between adolescence and early 20 s (*r* = −0.48, *p* = 0.014), suggesting a compensatory effect. In contrast, faster epigenetic aging between adolescence and late 20 s predicted faster epigenetic aging between adolescence and early 20 s (*r* = 0.55, *p* = 0.004) as well as between the early and late 20 s (*r* = 0.47, *p* = 0.015). These relationships are illustrated in Figure 2.

**FIGURE 3:**
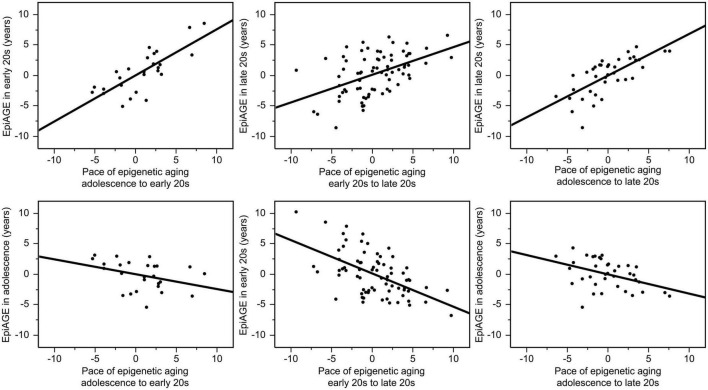
Correlations between the pace of epigenetic aging over a period and EpiAGE at the end **(top row)** as well as the beginning **(bottom row)** of the respective period. The pace of epigenetic aging between adolescence and the early 20 s was highly correlated with EpiAGE in the early 20 s (*r* = 0.77, *p* < 0.0001), the pace of epigenetic aging between the early 20 s and late 20 s was highly correlated with the EpiAGE in the late 20 s (*r* = 0.48, *p* < 0.0001), and the pace of epigenetic aging between adolescence and late 20 s was highly correlated with EpiAGE in the late 20 s (*r* = 0.71, *p* < 0.0001). In contrast, faster pace of epigenetic aging between early and late 20 s was negatively correlated with EpiAGE in the early 20 s (*r* = –0.55, *p* < 0.0001) and a faster pace of epigenetic aging between adolescence and the late 20 s was negatively correlated with EpiAGE in adolescence (*r* = –0.42, *p* = 0.008). The negative relationship between the faster pace of epigenetic aging between adolescence and the early 20 s and EpiAGE in adolescence (*r* = –0.36, *p* = 0.069).

There were also high correlations between the pace of epigenetic aging over a period and EpiAGE at the end of the respective period. The pace of epigenetic aging between adolescence and the early 20 s was highly correlated with EpiAGE in the early 20 s (*r* = 0.77, *p* < 0.0001), the pace of epigenetic aging between the early 20 s and late 20 s was highly correlated with the EpiAGE in the late 20 s (*r* = 0.48, *p* < 0.0001), and the pace of epigenetic aging between adolescence and late 20 s was highly correlated with EpiAGE in the late 20 s (*r* = 0.71, *p* < 0.0001). In contrast, more negative EpiAGE at the earlier timepoint, indicating younger epigenetic age, was associated with a faster subsequent pace of epigenetic aging, suggesting a compensatory effect. Faster pace of epigenetic aging between early and late 20 s was negatively correlated with EpiAGE in the early 20 s (*r* = −0.55, *p* < 0.0001) and a faster pace of epigenetic aging between adolescence and the late 20 s was negatively correlated with EpiAGE in adolescence (*r* = −0.42, *p* = 0.008). There was also a trend for a negative relationship between the faster pace of epigenetic aging between adolescence and the early 20 s and EpiAGE in adolescence (*r* = −0.36, *p* = 0.069). These relationships are illustrated in Figure 3.

### 3.2. Sex differences in the EpiAGE and the pace of epigenetic aging

The epigenetic aging was faster in young adult men than in young adult women and this medium-to-large effect of sex on the EpiAGE was present in both the late 20 s [*t*_(259)_ = 3.24, *p* = 0.001, Cohen’s *d* = 0.40] and the early 20 s [*t*_(74)_ = 2.94, *p* = 0.004, Cohen’s *d* = 0.70]. The effect of sex on EpiAGE in adolescence did not reach significance [*t*_(36)_ = −1.58, *p* = 0.123]. The effect of sex on EpiAGE in the late 20 s remained significant (beta = −0.19, *p* < 0.01) also when correcting the model for smoking, BMI and age. The effect of sex on EpiAGE in the early 20 s reduced to a trend (beta = −0.25, *p* = 0.07) when correcting the model for smoking, BMI and age, and remained insignificant also in adolescence (beta = 0.37, *p* = 0.07). There were no sex differences in the pace of epigenetic aging between the three different measurements (*p* > 0.115) and no sex differences in the pace of aging appeared (*p* > 0.06) also when correcting the model for smoking, BMI and age.

### 3.3. Does accelerated epigenetic aging predict accelerated brain aging or are these independent?

**FIGURE 4:**
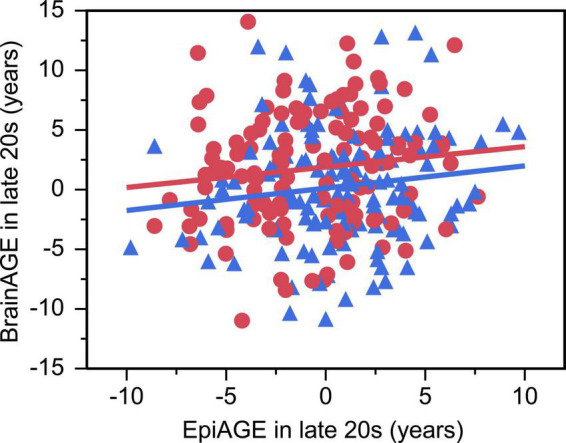
Accelerated epigenetic aging predicted accelerated brain aging in late 20 s (beta = 0.14, *p* = 0.032, Adj R^2^ = 0.03). Women are depicted as red circles and men as blue triangles.

There was a small but significant positive relationship between EpiAGE and BrainAGE in the larger sample of young adults in their late 20 s (beta = 0.14, *p* = 0.032, Adj *R*^2^ = 0.03, *n* = 261; Figure 4), which was not moderated by sex (beta = −0.006, *p* = 0.925). No similar relationship was found between EpiAGE and BrainAGE in the smaller sample of young adults in their early 20 s (beta = 0.05, *p* = 0.707, *n* = 76). The relationship between EpiAGE and BrainAGE in the larger sample of young adults in their late 20 s remained significant (beta = 0.13, *p* = 0.04, Adj *R*^2^ = 0.02, *n* = 261) when correcting the model for smoking, BMI and age and remained insignificant in the smaller sample of young adults in their early 20 s (beta = 0.07, *p* = 0.61, *n* = 76) when correcting the model for smoking, BMI and age.

### 3.4. Does accelerated epigenetic aging already predict deficits in cognition in young adulthood? And if so, do these deficits manifest in the verbal or performance IQ domain?

**FIGURE 5:**
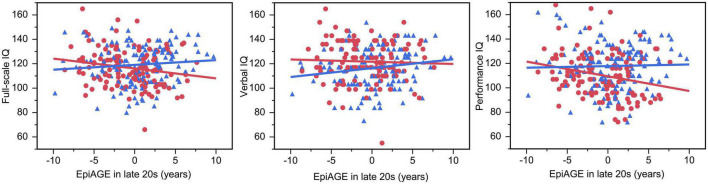
Accelerated epigenetic aging predicted lower full-scale IQ and performance IQ in women but not men. In women, higher EpiAGE was associated with lower full-scale IQ (beta = –0.19, *p* = 0.035, *R*^2^ = 0.04) and performance IQ (beta = –0.23, *p* = 0.009, *R*^2^ = 0.05) but not verbal IQ (beta = –0.04, *p* = 0.657). Women are depicted as red circles and men as blue triangles.

The GLM showed an interaction between EpiAGE in late 20 s and sex on full scale IQ (beta = −0.14, *p* = 0.023; Figure 5) and *post hoc* analyses revealed that higher EpiAGE was associated with lower full-scale IQ in women (beta = −0.19, *p* = 0.04, *R*^2^ = 0.04, *n* = 126) but not men (beta = 0.09, *p* = 0.282, *n* = 135). Further exploratory analyses aiming to determine whether these deficits manifest in the verbal or performance IQ domain showed that the sex-specific effects are pronounced in performance IQ (beta = −0.23, *p* = 0.009, *R*^2^ = 0.05) but not verbal IQ (beta = −0.04, *p* = 0.657). These interactions between EpiAGE and sex remained significant for the full scale IQ (beta = −0.13, *p* = 0.04) and performance IQ (beta = −0.12, *p* = 0.04) and remained insignificant for the verbal IQ (beta = −0.09, *p* = 0.15) when correcting the model for smoking, BMI and age.

## 4. Discussion

We performed a longitudinal study of epigenetic aging, brain aging, and cognitive skills in the ELSPAC prenatal birth cohort and showed that while our participants were tested at approximately the same chronological age in adolescence (*M* = 14.65 years, SD = 0.84), early 20 s (*M* = 23.85 years, SD = 0.39) and late 20 s (*M* = 29.49 years, SD = 0.64), their epigenetic age gap (EpiAGE) varied substantially and the range of the epigenetic age gap increased with age: from approximately ± 5 years in adolescence to approximately ± 10 years in the late 20 s. Still, the EpiAGE measures at different time points were positively correlated, suggesting that EpiAGE is a relatively stable characteristic of an individual across adolescence and early adulthood. The correlation between the closest EpiAGE measurements (e.g., early and late 20 s) was high, and the correlation between the more distant measurements (e.g., adolescence and late 20 s) was medium. These findings are consistent with Marioni et al. (2019) who tracked the Horvath epigenetic clock across the human life course and reported correlations between the EpiAGE measured at different time points between 0.22 to 0.82, with stronger associations in samples collected closer in time and concluded that the further measures have been more influenced by environmental factors. Our findings are also consistent with our previous work on brain age gap (BrainAGE) in the ELSPAC prenatal birth cohort (Mareckova et al., 2023), which also showed high correlation (*r* = 0.7) and thus very good stability of brain age gap in young adulthood.

Since our longitudinal dataset also allowed us to calculate the pace of epigenetic aging between the three different measurements, we additionally demonstrated that a faster pace of aging was positively correlated with EpiAGE at the end of the period (e.g., pace of aging between early 20 s and late 20 s and EpiAGE in late 20 s) but negatively correlated with EpiAGE at the earlier timepoint (e.g., pace of aging between early 20 s and late 20 s and EpiAGE in early 20 s). These findings suggest a possible existence of a compensatory mechanism where late maturers (e.g., those appearing epigenetically younger than their chronological age during the early measurements) are catching up (and thus experience faster pace of epigenetic aging) with the early maturers (e.g., those appearing epigenetically older than their chronological age during the early measurements). These results are also consistent with our other findings of the negative relationship between (1) the pace of aging between adolescence and the early 20 s and (2) the pace of aging between the early and late 20 s.

Consistently with Horvath et al. (2016), we also found greater positive EpiAGE in men vs. women, suggesting faster epigenetic aging in men. In our study, this effect was large in the early 20 s and medium in the late 20 s. Kankaanpää et al. (2022) conducted a twin study testing the mechanisms underlying sex differences in epigenetic aging and found that several lifestyle-related factors, including smoking and BMI, partly mediated the association of sex with biological aging. However, since sex differences in epigenetic age acceleration were also reported in adolescents, children, and newborns (Simpkin et al., 2016), other mechanisms are also likely contributing to the sex differences in epigenetic aging and the sex morbidity-mortality paradox, according to which women possess a lower age-adjusted mortality rate compared to men (Case and Paxson, 2005; Oksuzyan et al., 2008).

Our study also pointed out that while a higher positive epigenetic age gap predicted a higher positive brain age gap assessed on the same day in the late 20 s, this effect was small (Adj *R*^2^ = 0.03) and did not reach significance in a smaller sample of participants in their early 20 s. This small effect size is consistent with research from others who also found only weak associations between brain age and methylation age (Cole et al., 2019; Teeuw et al., 2021; Zheng et al., 2022). Still, it supports the research by Lu et al. (2017) and Hillary et al. (2021) who reported associations between neuron density and methylation age in older adults. On the other hand, the discrepancies between these two measures of aging might be potentially explained by the fact that DNA methylation is a measure of cellular aging (Lowe et al., 2016; Kabacik et al., 2018), the gradual decline in cell function, but aging of the brain, described by lower cortical thickness, is rather due to cellular senescence (Fernandez-Egea and Kirkpatrick, 2017), the cessation of cell division.

The relationship between the higher positive epigenetic age gap and lower full-scale IQ in young adulthood had a very similar effect size (Adj *R*^2^ = 0.03) but was significant in women only. This sex-specific effect was driven by the performance IQ (Adj R^2^ = 0.05) and not verbal IQ. These findings suggest that despite the overall lower speed of epigenetic aging in women vs. men (Horvath et al., 2016; Simpkin et al., 2016; Kankaanpää et al., 2022) discussed above, women might be more vulnerable to the negative impact of aging on cognition and that the performance IQ domain might serve as an early marker of cognitive decline. These findings are broadly consistent with Levine et al. (2021) who studied more than 26 000 individuals from 5 prospective cohort studies and concluded that women have higher baseline performance in global cognition, executive function and memory than men, but significantly faster decline in global cognition, executive function but not memory. Our findings are also broadly consistent with those of Zheng et al. (2022) who studied cognitive skills and their relationship with epigenetic and brain aging in midlife. They showed that both faster epigenetic and brain aging were associated with worse cognitive skills and particularly the score on the Stroop task, which evaluates the ability to respond to one stimulus and suppress the response to another, an executive skill attributed to the frontal lobe; the Rey Auditory Verbal Learning Test (RAVLT), which evaluates one’s verbal memory; and the Digit Symbol Substitution Test (DSST), which evaluates visual-motor speed, sustained attention and working memory (Zheng et al., 2022). While the DSST test, also known as digit-symbol coding, is part of the WAIS performance IQ test used in the current study, the Stroop and RAVLT tasks are not part of the WAIS IQ test used in the current study, suggesting that accelerated epigenetic aging might also contribute to individual differences in executive function and verbal memory.

Our study has several limitations, including the relatively small sample size of epigenetic data from adolescence and early adulthood and the fact that the IQ was assessed in the late 20 s but not in the early 20 s. Further, our DNA samples were isolated from saliva, allowing us to calculate the epigenetic age based on Horvath’s multi-tissue epigenetic clock, but future research might consider collecting blood samples and replicating our findings using blood-based epigenetic clocks such as the Hannum et al. (2013) or GrimAGE (Lu et al., 2019). Finally, while all the samples were analyzed using the same type of chip, the samples from adolescence and early 20 s were collected and analyzed earlier than the samples from the late 20 s and therefore the three samples per individual could not be placed on the same chip. Therefore, we have presented the results for each period separately. Still, the longitudinal design of our prenatal birth cohort study with three epigenetic and two neuroimaging assessments allowed us to calculate not only EpiAGE and BrainAGE at different time points but also the pace of aging between the time points, providing a unique contribution to the literature. Moreover, our analyses used sex not only as a covariate as in the research by others (Zheng et al., 2022) but evaluated the potential moderating effects of sex. We conclude that the epigenetic age gap is a relatively stable characteristic of an individual across adolescence and early adulthood, that faster pace of epigenetic aging between two measurements predicts higher EpiAGE, suggesting accelerated epigenetic aging at the end of the period, and that accelerated epigenetic aging shows a small but significant relationship with accelerated brain aging and women-specific relationship with worse performance IQ.

## Data availability statement

The datasets presented in this article are not readily available due to ethical restrictions, in order to protect participant privacy. Requests to access the datasets should be directed to the corresponding author.

## Ethics statement

The studies involving human participants were reviewed and approved by the ELSPAC Ethics Committee, Masaryk University. The patients/participants provided their written informed consent to participate in this study.

## Author contributions

KM: conceptualization, formal analysis, writing—original draft, visualization, and funding acquisition. AP: methodology and software. RM: methodology and software. LS: investigation and data curation. LI: investigation and resources. JK: resources and funding acquisition. MB: funding acquisition. YN: supervision and writing—review and editing. All authors contributed to the article and approved the submitted version.
