# Supplementary material for: Longitudinal study of epigenetic aging and its relationship with brain aging and cognitive skills in young adulthood
Source: Front Aging Neurosci. 2023 Aug 1;15:1215957. doi: 10.3389/fnagi.2023.1215957 (PMC10427722; doi:10.3389/fnagi.2023.1215957)
Supplement: Supplementary file 1 [file Table_1.DOCX]

**Supplementary Table 1 – Demographics Table**

|  | **Adolescence**  (n=39) | **Early 20s**  (n=76) | **Late 20s**  (n=261) |
| --- | --- | --- | --- |
| **Sex**  % Men | 55% | 58% | 52% |
| **Ethnicity**  % White Caucasian | 100% | 100% | 100% |
| **Age**  in years (M, SD) | M=14.65, SD=0.84 | M=23.85, SD=0.39 | M=29.49, SD=0.64 |
| **BMI** (M, SD) | NA | M=23.12, SD=3.39 | M=24.26, SD=4.03 |
| **Smoking**  % not at all  % less than once a week  % less than once a day  % daily | NA | 76%  5%  6%  13% | 79%  5%  4%  12% |
| **Birth Weight**  in grams (M, SD) | M=3352.78, SD=465.98 | M=3313.68, SD=513.32 | M=3313.68, SD=513.32 |
| **Maternal Age at Birth**  in years (M, SD) | M=27.56, SD=3.86 | M=27.54, SD=4.88 | M=27.54, SD=4.88 |
