# Supplementary figures and images for: Longitudinal study of epigenetic aging and its relationship with brain aging and cognitive skills in young adulthood
Source: Front Aging Neurosci. 2023 Aug 1;15:1215957. doi: 10.3389/fnagi.2023.1215957 (PMC10427722; doi:10.3389/fnagi.2023.1215957)

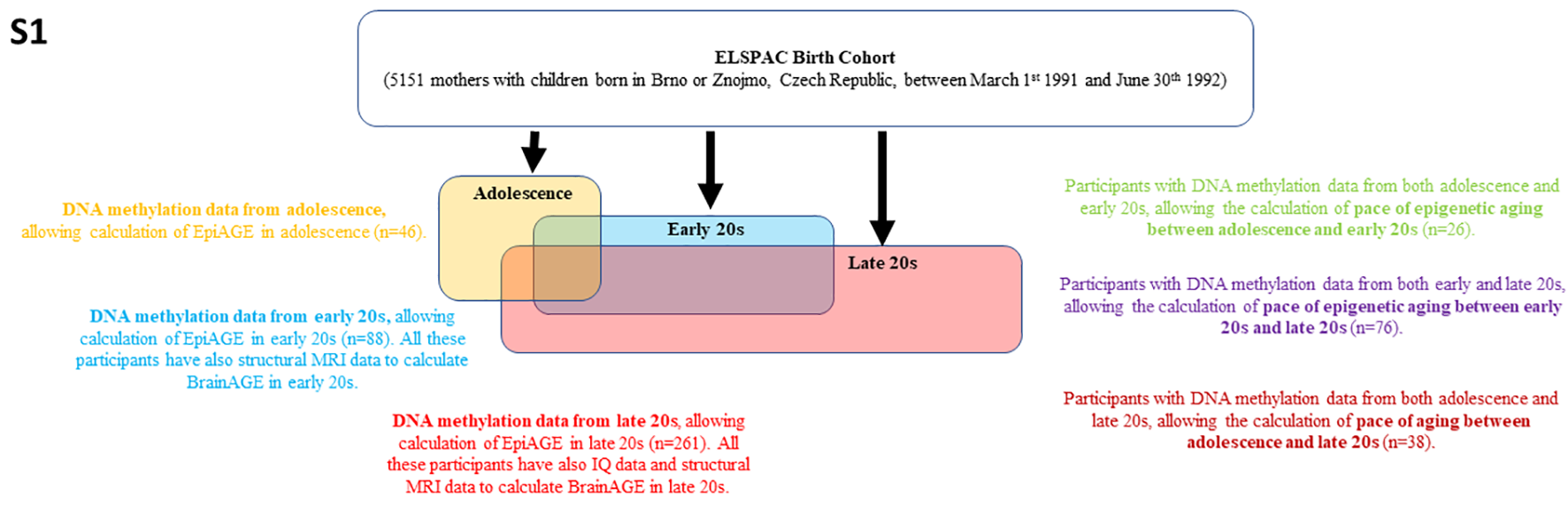

Supplement: Supplementary file 2 [file Image_1.TIF]
